# Membranous and nuclear staining of CLDN18 in HPV‐independent and HPV‐associated endocervical adenocarcinomas

**DOI:** 10.1002/cam4.5029

**Published:** 2022-07-21

**Authors:** Xiuzhen Du, Yanjiao Hu, Xiaoyu Ji, Lei Sui, Qingmei Zheng, Kejuan Song, Teng Lv, Yulong Chen, Han Zhao, Shuzhen Dai, Peng Zhao, Qin Yao

**Affiliations:** ^1^ Department of Obstetrics and Gynecology The Affiliated Hospital of Qingdao University Qingdao Shandong China; ^2^ Department of Pathology The affiliated Hospital of Qingdao University Qingdao Shandong China

**Keywords:** CLDN18, endocervical adenocarcinoma, HPV, immunohistochemistry, membranous expression, nuclear expression

## Abstract

**Objectives:**

A classification system for endocervical adenocarcinoma (ECA) based on high‐risk human papillomavirus (HPV) status has been established; however, the immunohistochemical markers distinguishing HPV‐independent and HPV‐associated ECAs have not been fully described. Here, we aimed to characterize ECA immunopathological features.

**Methods:**

We evaluated the immunohistochemical profile of CLDN18, CDX2, PAX8, p16, p53, and CEA in 60 ECAs comprising 10 HPV‐independent ECAs and 50 HPV‐associated ECAs. Both the membranous and nuclear expression levels of CLDN18 were analyzed.

**Results:**

Membranous CLDN18 (CLDN18 [M]) was found to be expressed in the mucinous epithelium of all HPV‐independent ECAs, including eight gastric‐type ECAs (G‐ECAs), one endometrioid ECA, and one clear cell ECA, but no nuclear CLDN18 (CLDN18 [N]) expression was detected in HPV‐independent ECAs. Among HPV‐associated ECAs, CLDN18 (M) expression levels in intestinal‐type (I‐ECAs) and usual‐type ECAs (U‐ECAs) were significantly different from those in invasive stratified mucin‐producing (iSMILE) carcinomas (*p* = 0.036). Positive CLDN18 (M) staining was present in 55.6% (5/9) of intestinal‐type and 39.4% (13/33) of usual‐type ECAs and was not present in iSMILE ECAs. Silva pattern C cancers expressed higher levels of CLDN18 (M) than Silva pattern A and B cancers (*p* = 0.004), whereas the CLDN18 (N) expression levels in cancers showing Silva pattern A were significantly higher than those in cancers exhibiting Silva patterns B and C (*p* < 0.001).

**Conclusion:**

Membranous CLDN18 is expressed in ECAs and is particularly frequently expressed in HPV‐independent ECAs, and membranous CLDN18 expression has potential as a therapeutic target. Nuclear staining of CLDN18 is a new immunohistochemical marker for diagnosing Silva pattern A HPV‐associated ECAs and is associated with a good prognosis. Further studies should investigate the therapeutic and prognostic significance of membranous and nuclear CLDN18 expression and develop a related test that can be implemented in the clinical evaluation of ECAs.

## Graphical abstract

Membranous claudin‐18 expression is expressed in human papillomavirus (HPV)‐independent endocervical adenocarcinomas (ECAs), particularly gastric‐type ECAs. Membranous claudin‐18 expression indicates its potential as a therapeutic target for ECAs. Nuclear staining of claudin‐18 is a new immunohistochemical marker for diagnosing Silva A pattern in HPV‐associated ECAs.

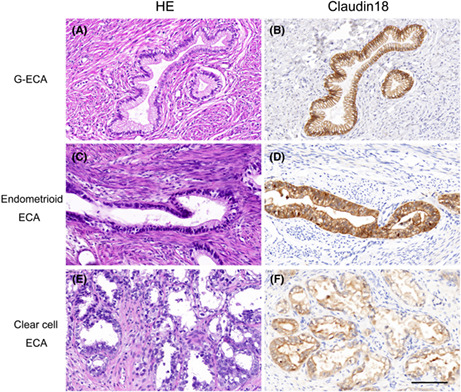

## 1 INTRODUCTION

Endocervical adenocarcinoma (ECA) accounts for 20%–25% of cervical cancers, and its incidence is increasing relative to that of cervical squamous cell carcinoma (SQCC) and in absolute numbers,^1^, ^2^, ^3^ especially in the era of the HPV vaccine. According to the International Endocervical Adenocarcinoma Criteria and Classification (IECC), it is divided into two categories, HPV‐associated ECA and HPV‐independent ECA.^4^ ECAs are considered to be heterogeneous tumors with different etiologies, pathogenesis mechanisms, molecular features, treatment responses, and prognoses.^5^ It has been suggested that HPV‐independent ECA is associated with a poorer prognosis than HPV‐associated ECA.^6^, ^7^, ^8^ Among the HPV‐independent ECAs, the gastric type is most common; however, other types (endometrioid adenocarcinoma, clear cell adenocarcinoma, serous adenocarcinoma, and mesonephric carcinoma) are rare.^6^

CLDN18 is an adhesion molecule extensively expressed in the cytoplasmic membrane of gastric epithelial cells in normal and gastric cancer tissue. CLDN18 is expressed along the basolateral membrane in all types of differentiated gastric epithelial cells, including surface mucous cells, chief cells, parietal cells, and endocrine cells.^9^ Therefore, membranous CLDN18 is considered to be a highly selective immunohistochemical marker to detect gastric differentiation in pancreatobiliary,^10^ colorectal,^11^ and ovarian mucinous neoplasms.^9^, ^12^

The CLDN family comprises at least 27 transmembrane proteins that are the main components of tight junctions (TJs). CLDNs generally localize to the apical and basolateral regions of the cell membrane and play an important role in cell–cell adhesion, maintenance of cell polarity and selective paracellular permeability, regulating epithelial‐mesenchymal transformation (EMT), cell proliferation, migration and infiltration in cancer. On the other hand, claudins have one putative or multiple nuclear localization sequences (NLSs) that facilitate transport into the nucleus. Nuclear CLDNs interact with transcriptional regulators that impact gene expression and the regulation of cell apoptosis.^13^ Recently, nuclear staining of CLDN18 was shown to be a new immunohistochemical marker for diagnosing intramucosal well‐differentiated gastric adenocarcinoma.^14^

A few studies have shown membranous immunohistochemical expression of CLDN18 in endocervical lesions, such as gastric‐type adenocarcinoma in situ, gastric‐type ECAs and nongastric‐type ECAs.^15^, ^16^, ^17^ No relevant studies have investigated CLDN18 and its different subcellular locations in HPV‐independent and HPV‐associated ECAs. Moreover, diagnostic classification of ECAs has been a significant challenge due to their histopathologically similar morphology, especially when only a limited amount of biopsy material is examined, suggesting that a series of immunohistochemical markers is required to improve diagnostic accuracy.^18^ Therefore, we conducted the present study to evaluate the membranous and nuclear expression levels of CLDN18 and their association with clinicopathological and immunophenotypic parameters in HPV‐independent and HPV‐associated ECAs.

## 2 MATERIALS AND METHODS

### 2.1 Patient selection

This study was performed in accordance with the institutional review board approval from the medical ethical committee of the Affiliated Hospital of Qingdao University. There were 62 patients who had undergone radical hysterectomy or hysterectomy and were histologically diagnosed with ECA at West Coast Hospital and Shinan District Hospital, the Affiliated Hospital of Qingdao University, between 2014 and 2019. All formalin‐fixed paraffin‐embedded (FFPE) tissue specimens were selected from the pathology archives at the Department of Pathology. All whole hematoxylin and eosin (HE)‐stained sections were reviewed by two pathologists, and the histological subtypes were classified according to the 2020 WHO classification of tumors of the uterine cervix and the International Endocervical Adenocarcinoma Criteria and Classification (IECC) system.^16^ Two patients with no adequately suitable specimens for immunohistochemical analysis were excluded. There were no patients with mesonephric or serous ECA. The 60 patients (age range, 31–81 years; median age, 51 years) comprised 33 patients with usual‐type endocervical adenocarcinomas (U‐ECAs), nine patients with intestinal‐type endocervical adenocarcinomas (I‐ECAs), eight patients with invasive stratified mucin‐producing carcinomas (iSMILE‐ECAs), eight patients with gastric‐type endocervical adenocarcinomas (G‐ECAs), one patient with clear cell adenocarcinoma and one patient with endometrioid adenocarcinoma. Normal endocervical glands were found in 17 of the 60 patients. Furthermore, 10 normal endocervical glandular mucosae (NEM) samples and 20 cervical SQCC samples were evaluated.

### 2.2 Immunohistochemistry analysis

Serial sections of 4 mm thickness were taken from representative FFPE tissue blocks, affixed to 3‐aminopropyl triethoxysilane‐coated slides, and air‐dried overnight at 37°C. The sections were then subjected to HE and immunohistochemical staining. Immunohistochemical staining was performed using the following immunoperoxidase polymer methods. After dewaxing and antigen retrieval, endogenous peroxidase activity was blocked with 0.3% hydrogen peroxide for 10 min. After blocking with goat serum, the sections were incubated for 30 min at 37°C with rabbit anti‐CLDN18 (1:500 dilution, clone ab203563; Abcam), rabbit anti‐CDX2 (1:50 dilution, clone A20222; ABclonal Technology Co), rabbit anti‐PAX8 (1:100 dilution, clone bs‐1201R; BIOSS ANTIBODIES), mouse anti‐CEA (1:300 dilution, clone 12‐140‐10; ZSGB‐BIO), mouse anti‐p53 (1:300 dilution, clone DO‐7; ZSGB‐BIO), and mouse anti‐P16 (1:100 dilution; clone MX007, MXB). The slides were visualized with diaminobenzidine (DAB) and counterstained with hematoxylin. Both membranous and nuclear CLDN18 levels were evaluated. The stained slides were scanned with a Pannoramic SCAN slide scanner (3DHISTECH Ltd.) to obtain a whole slide image.

### 2.3 Immunohistochemical evaluation

CDX2, PAX8, CEA, and CLDN18 expression were assessed using a semiquantitative pathology H‐score, according to staining intensity and percent of corresponding intensity tumor cells. The intensity was scored as follows: No staining as 0, weak staining as 1+, moderate staining as 2+, and strong staining as 3+. In brief, the H‐score was calculated as follows: (Percentage of expression 1+ tumor cells) × 1 + (percentage of expression 2+ tumor cells) × 2 + (percentage of expression 3+ tumor cells) × 3. The score can range from 0 to 300. In this study, a cutoff of 50 was used, and scores were categorized as negative or low (0–50) and positive or high (51–300).^19^, ^20^ The criterion for subclonal CLDN18 was defined as abnormal staining of more than 12 consecutive cells on the background of abnormal CLDN18 staining.^21^ P16 was evaluated as positive with diffuse, block‐like expression, whereas no or patchy expression was negative.^4^ P53 expression was considered aberrant when it was overexpressed, completely absent in the nucleus, or expressed in the cytoplasm.^22^

### 2.4 High‐risk HPV DNA assay

For high‐risk HPV testing, each sample was evaluated using the Roche Cobas® 4800 real‐time PCR HPV test. The assay detects 14 high‐risk types, with individual genotyping for HPV16 and HPV18, and the other 12 types were reported by a pooled result.^23^

### 2.5 Silva system

Invasive ECAs were classified based on the pattern of invasion (Pattern A, B or C).^24^ Briefly, Pattern A is characterized by well‐demarcated glands without destructive stromal invasion or lymphovascular invasion. Pattern B is characterized by early destructive stromal invasion arising from otherwise well‐demarcated glands and can present with lymphovascular invasion. Pattern C is characterized by extensive and diffuse destructive stromal invasion.

### 2.6 Statistical analysis

The differences and associations between immunohistochemical results and clinicopathological features were compared using the *χ* ^2^ test and Fisher's exact test, with statistical significance at *p* < 0.05.

## 3 RESULTS

### 3.1 CLDN18 expression in ECAs

**FIGURE 1:**
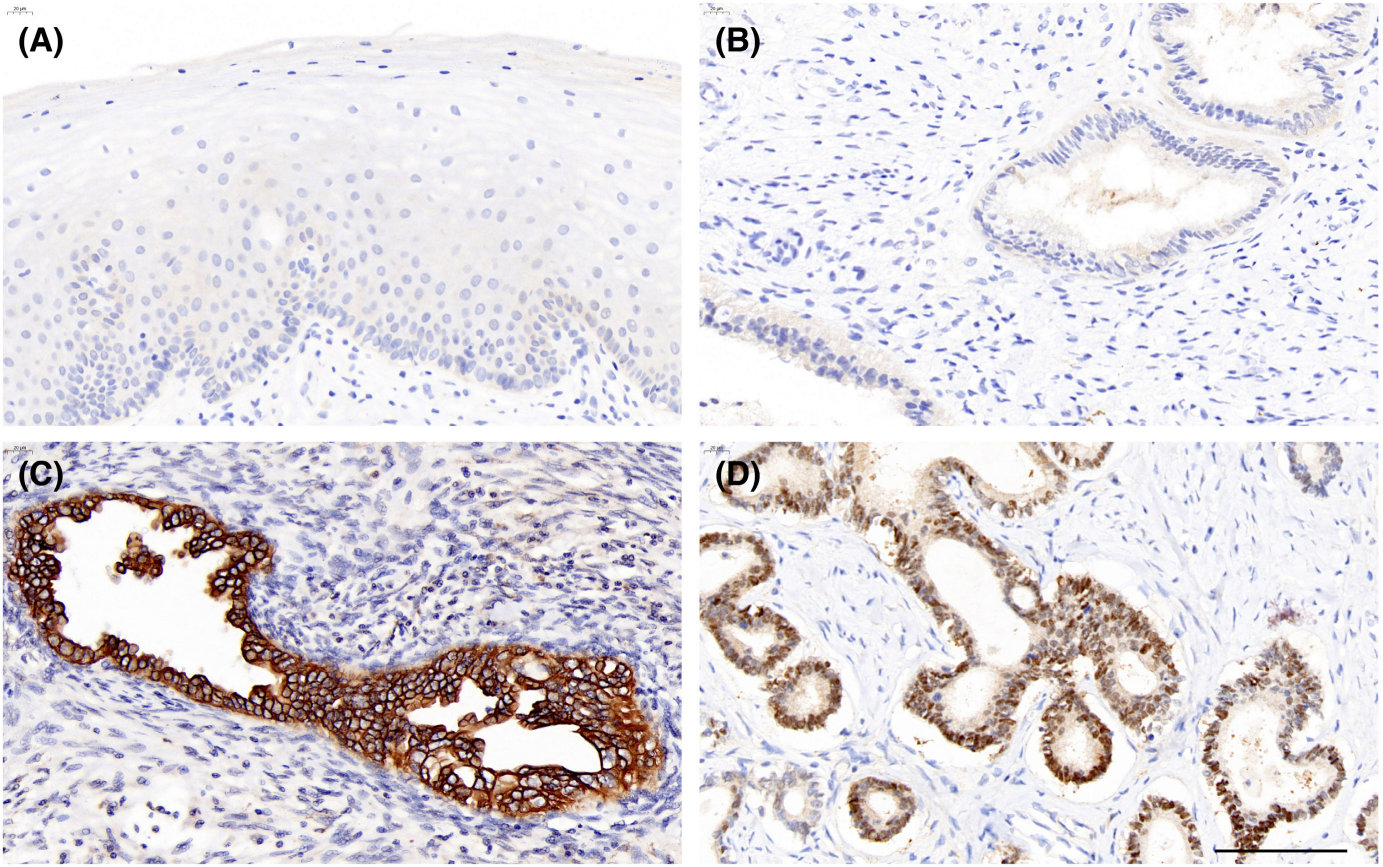
Different subcellular locations of CLDN18 expression in ECAs. (A, B) Nonneoplastic cervical epithelial cells, columnar epithelium and squamous epithelium are negative for CLDN18 expression. (C) ECA sample positive for CLDN18 (M) expression. (D) ECA sample positive for CLDN18 (N) expression (bar = 100 μm). ECA, endocervical adenocarcinoma.

Nonneoplastic cervical epithelial cells, columnar epithelium and squamous epithelium did not express CLDN18. Cancer cells were considered CLDN18‐positive if membranous or nuclear staining was present. Overall, CLDN18 (M) expression was present in 46.7% (28/60) of cases, 11 with membranous staining and 17 with mixed cytoplasmic/membranous staining. Of note, 14 primary cases (23.3%) demonstrated CLDN18 (N) expression (6 with nuclear expression and 8 with mixed nuclear/cytoplasmic expression) (Figure 1).

### 3.2 CLDN18 expression in HPV‐independent ECAs

**FIGURE 2:**
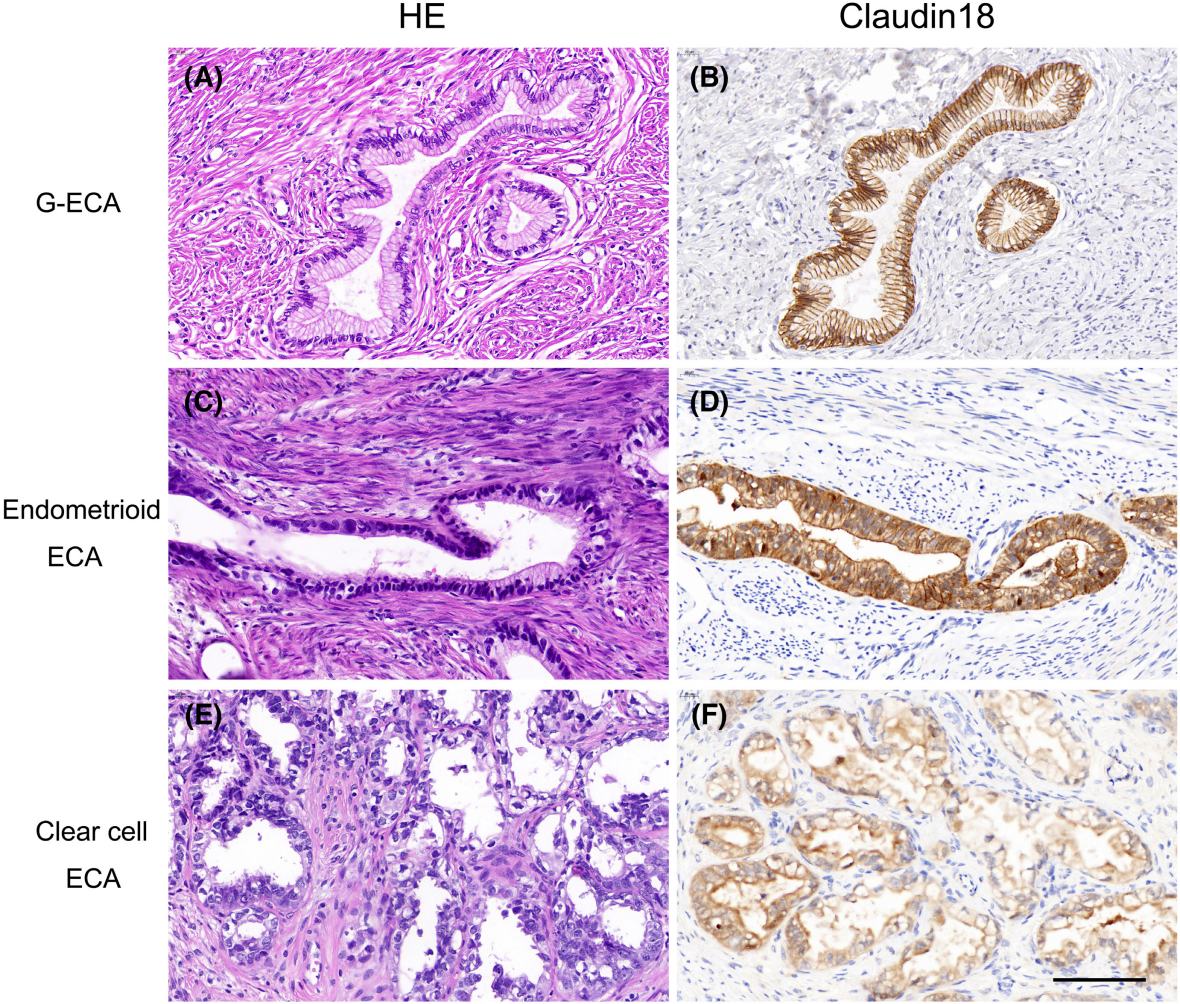
CLDN‐18 expression in HPV‐independent ECAs. (A) Gastric‐type ECA (HE); (B) strong CLDN18 (M) staining. (C) Endometrioid ECA (HE); (D) strong CLDN18 (M) staining. (E) Clear cell ECA (HE); (F) weak CLDN18 (M) staining (bar = 100 μm). ECA, endocervical adenocarcinoma; HE, hematoxylin and eosin; HPV, human papillomavirus.

All 8 G‐ECAs were positive for CLDN18 (M) expression in the mucinous epithelium, with seven showing strong staining patterns and one exhibiting moderate staining. One endometrioid ECA and one clear cell ECA were strongly and weakly positive for CLDN18 (M), respectively (Figure 2). However, no CLDN18 (N) expression was detected in HPV‐independent ECAs.

### 3.3 CLDN18 expression and clinicopathological associations in HPV‐associated ECAs

In HPV‐associated ECAs, CLDN18 (M) was expressed in the mucinous epithelium of numerous ECAs (18/50 cases, 36.0%). CLDN18 (M) expression positivity showed a statistically significant association with histological type and was significantly higher in intestinal and usual‐type ECAs than in iSMILE‐type ECAs (*p* = 0.036). Positive CLDN18 (M) staining was present in 5/9 (55.6%) and 13/33 (39.4%) intestinal‐type and usual‐type ECAs, respectively, and was not present in iSMILE‐ECAs. Furthermore, high CLDN18 (M) expression was also associated with the Silva pattern and stromal invasion, and a higher incidence of positive cases was found among the Silva pattern C (*p* = 0.004) and deep stromal invasion (*p* = 0.016) groups. CLDN18 (M) expression was not significantly associated with age, grade, stage, lymph node metastases, nerve invasion, or lymphovascular space involvement (LVSI) (Table 1).

**TABLE 1:** Clinicopathological features of the considered series according to claudin‐18 (M) and claudin‐18 (N) status in HPV‐associated ECAs

Characteristic	*n*	Claudin‐18 (M) *n* (%)	*p*	Claudin‐18 (N) *n* (%)	*p*
Age (years)					
<50 years	24	10 (41.7)	NS	5 (20.8)	NS
≥50 years	26	8 (30.8)		9 (34.6)	
Histologic grade					
G1/2	44	15 (34.1)	NS	12 (27.3)	NS
G3	6	3 (50.0)		2 (33.3)	
Stage					
I	40	12 (30.0)	NS	14 (35.0)	NS
II	2	2 (100)		0 (0)	
III	6	2 (33.3)		0 (0)	
IV	2	2 (100)		0 (0)	
Lymph node metastases					
Positive	6	2 (33.3)	NS	2 (33.3)	NS
Negative	44	16 (36.4)		12 (27.3)	
LVSI					
Positive	6	2 (33.3)	NS	4 (66.7)	NS
Negative	44	16 (36.4)		10 (22.7)	
Nerve invasion					
Positive	2	2 (100)	NS	0 (0)	NS
Negative	48	16 (33.3)		14 (29.2)	
Stromal invasion					
≤1/2	29	6 (20.7)	0.016	9 (32.1)	NS
>1/2	21	12 (57.1)		5 (22.7)	
Sillva type					
A	14	1 (7.1)	0.004	10 (71.4)	<0.001
B	6	1 (16.7)		2 (33.3)	
C	30	16 (53.3)		2 (6.7)	
Histologic type					
I‐EAC	9	5 (55.6)	0.036	0 (0)	NS
EAC‐iSMILE	8	0 (0)		2 (25.0)	
U‐EAC	33	13 (39.4)		12 (36.4)	

Abbreviations: ECA‐iSMILE, invasive stratified mucin‐producing carcinoma; I‐ECA, intestinal‐type ECA; U‐ECA, usual‐type ECA.

Of note, among 50 HPV‐associated ECAs, 14 primary cases (28.0%) demonstrated CLDN18 (N) expression. High CLDN18 (N) expression was associated with the Silva pattern, and a higher incidence of positive cases was found in the Silva pattern A group (*p* < 0.001). CLDN18 (N) expression was not significantly associated with age, grade, stage, or pathological type. Furthermore, CLDN18 (N) expression was not associated with lymph node metastases, nerve invasion, stromal invasion, or LVSI (Table 1).

**FIGURE 3:**
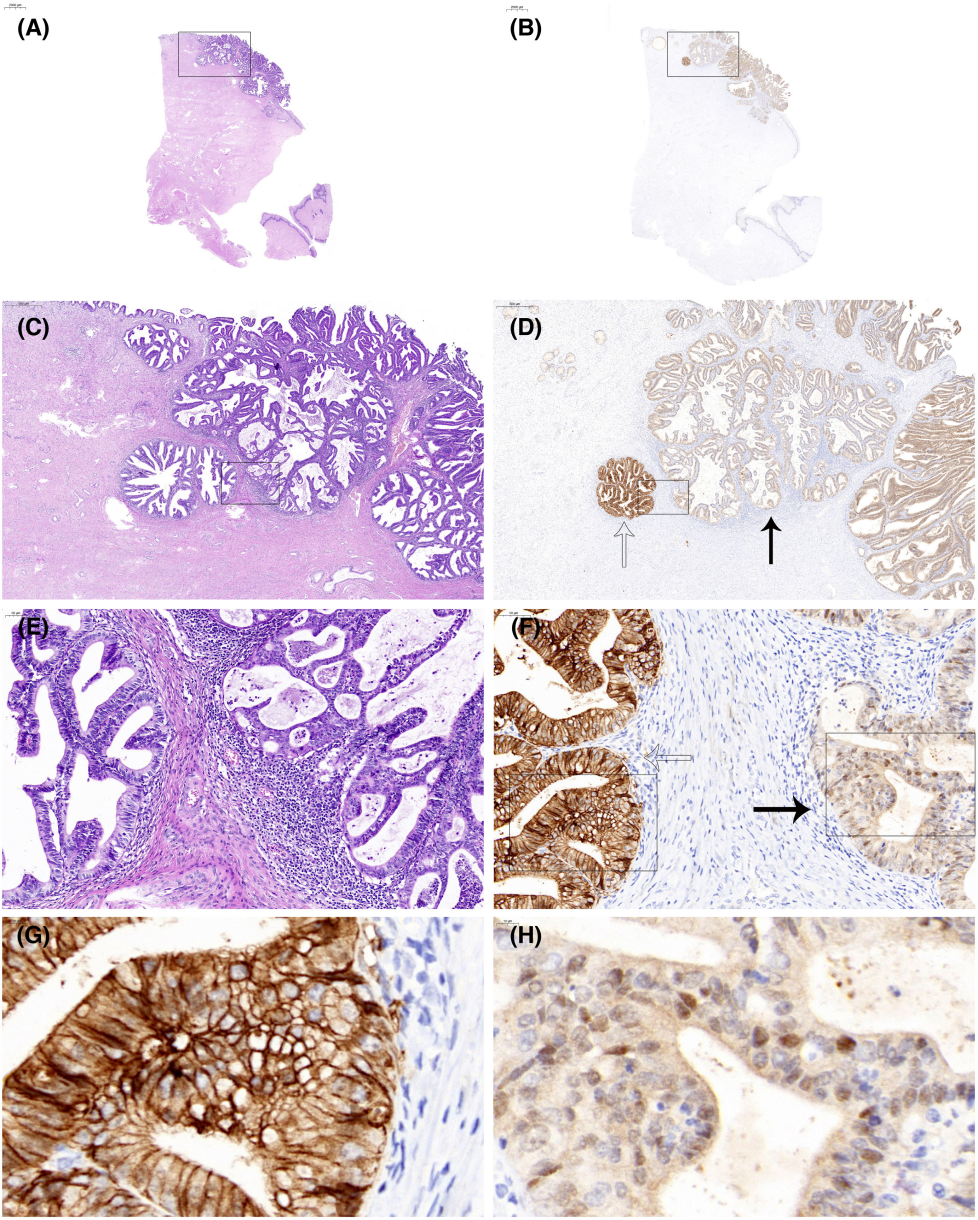
Usual‐type ECA with heterogeneous or subclonal CLDN18 expression. (A, C, E) HE staining at different low‐ and high‐power views. (B) Low‐power view shows CLDN18 expression in the ECA. (D) Areas with different CLDN18 staining intensities were admixed in the same tumor focus (strong expression at the hollow arrow and weak expression at the solid arrow). (F) Areas with different subcellular localizations of CLDN18 expression were admixed in the same tumor focus (region at the hollow arrow is mainly located in the membrane and cytoplasm, and the solid arrow indicates nuclear and cytoplasmic localization). (G) In some areas, CLDN18 expression is strong and located in the cell membrane. (H) In other areas, CLDN18 expression is weak and shows nuclear and cytoplasmic localization. ECA, endocervical adenocarcinoma; HE, hematoxylin and eosin.

Interestingly, six tumors (12.0%) showed concomitant nuclear and membranous CLDN18 expression within subclonal areas. Tumors were considered heterogeneous for CLDN18 if subclonal patterns exhibited different patterns of positive staining with membranous or nuclear CLDN18 in one whole section. Even areas with different staining intensities and distinct subcellular localization of CLDN18 staining were observed within the same tumor focus (Figure 3). Among 50 HPV‐associated ECAs, CLDN18 subclonal expression patterns were seen in two intestinal‐type (2/9, 22.2%) and 4 usual‐type cases (4/33, 12.1%). In the whole tumor sections, all 6 cases showed more than one subclonal area, and in 3/6 cases, subclonality was focal (<10%).

### 3.4 Expression of immunohistochemical markers in different ECA subtypes

**FIGURE 4:**
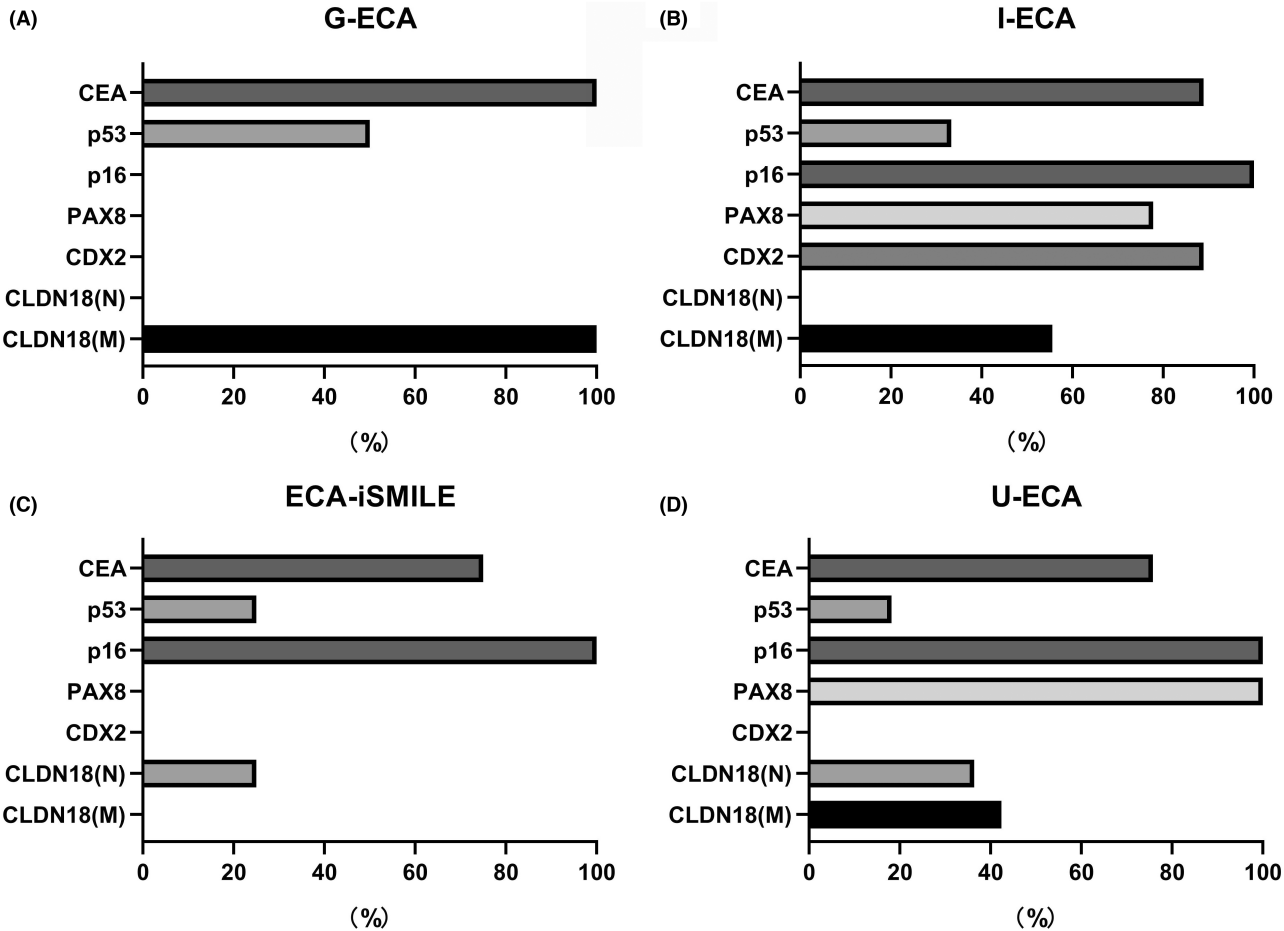
Expression of immunohistochemical markers in different ECA subtypes. Each graph shows the positive or aberrant expression rates of CDX2, PAX8, p16, p53, CEA, CLDN18 (M), and CLDN18 (N). (A) Gastric‐type ECA (*n* = 8). (B) Intestinal‐type ECA (*n* = 9). (C) iSMILE‐type ECA (*n* = 8). (D) Usual‐type ECA (*n* = 33). ECA, endocervical adenocarcinoma.

Among 60 ECA lesions, 33 (55.0%) were usual‐type, eight (13.3%) were gastric‐type, nine (15.0%) were intestinal‐type, and eight (13.3%) were iSMILE‐type. Figure 4 illustrates the immunohistochemical marker expression of CDX2, PAX8, p16, p53, and CEA, as well as CLDN18 (M) and CLDN18 (N), in different subtypes. As shown in Figure 4, CDX2 was expressed more frequently in intestinal‐type cancers than in gastric‐, usual‐ and iSMILE‐type cancers (*p* < 0.001). PAX8 expression was significantly lower in gastric‐ and iSMILE‐type cancers than in intestinal‐ and usual‐type cancers (*p* < 0.001). CLDN18 (M) was expressed in all gastric‐type cancers and tended to be expressed more often in intestinal‐ and usual‐type cancers than in iSMILE‐type cancers (*p* < 0.001). P16 was expressed in HPV‐associated intestinal‐type, usual‐type, and iSMILE‐type ECAs but was not expressed in HPV‐independent gastric‐type ECAs (*p* < 0.001). The rates of abnormal P53, CEA, and claudin (N) expression were not significantly different among the four subtypes.

**FIGURE 5:**
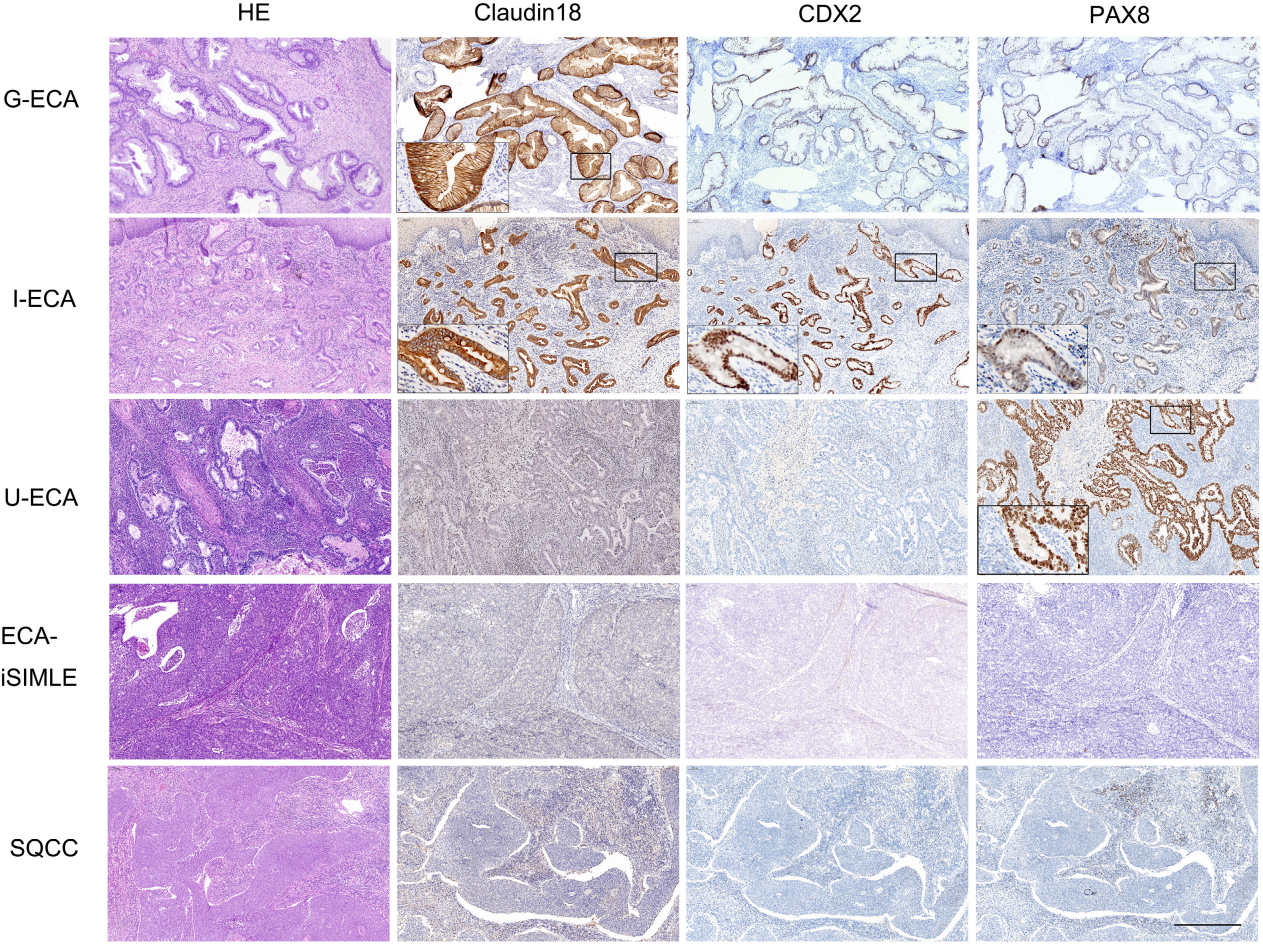
Immunohistochemical expression profiles of CLDN18 (M), CDX2, and PAX8 in G‐ECA, I‐ECA, U‐ECA, ECA‐iSMILE, and SQCC. The tumor cells of the I‐ECA exhibited gastric differentiation characterized by CLDN18 (M)‐positivity. ECA‐iSMILE, invasive stratified mucin‐producing carcinoma; G‐ECA, gastric‐type ECA; I‐ECA, intestinal‐type ECA; SQCC, squamous cell carcinoma (bar = 500 μm); U‐ECA, usual‐type ECA.

Based on the morphological features and results of the immunohistochemical analysis, we combined three phenotypic markers, claudin 18 (M) as gastric differentiation, CDX2 as intestinal differentiation, and PAX8 as Müllerian differentiation. We subclassified the ECAs as follows: Gastric‐type ECA, positive CLDN18 (M), and negative CDX2 expression; intestinal‐type ECA, positive CDX2 and positive PAX8 expression; usual‐type ECA, positive PAX8 and negative CDX2 expression; and iSMILE‐type ECA, negative expression of all three markers (CLDN18 [M], CDX‐2 and PAX8). The representative histological and immunohistochemical features of each ECA subtype are shown in Figure 5.

### 3.5 Gastric differentiation in intestinal‐type and usual‐type ECAs

Gastric differentiation characterized by CLDN18 (M) expression was observed in the mucinous epithelium of intestinal‐type and usual‐type ECAs. In a subset of usual‐type ECA cases (13/33, 39.4%), the tumor comprised mucinous epithelium exhibiting an intermediate gastric and usual phenotype and diffuse positive CLDN18 (M) and PAX8 expression. In another subset of intestinal‐type ECA cases (5/9, 55.6%), the tumor comprised mucinous epithelium exhibiting an intermediate gastric and intestinal phenotype and diffuse positive CLDN18 (M) and CDX2 expression. There was no CLDN18 (M) expression in iSMILE ECAs. Representative histological and immunohistochemical features with gastric differentiation in the intestinal subtype are shown in Figure 5.

## 4 DISCUSSION

In our study, the presence of CLDN18 (M) was documented in HPV‐independent ECA primary cases and intestinal‐type or usual‐type ECA with gastric differentiation. In addition to 10 HPV‐independent ECAs positive for CLDN18 (M) expression, of the 33 usual ECAs, 13 cases (39.4%) showed CLDN18 (M) expression, and CLDN18 (M) expression was present in 5 of 9 I‐ECA cases (55.6%). Similar expression with a gastric differentiation phenotype has been previously reported in a variety of tumors, including pancreatobiliary tumors,^10^, ^25^ ovarian mucinous tumors,^9^, ^12^ and endocervical adenocarcinomas.^16^, ^17^ Moreover, we found a significant association between CLDN18 (M) expression and Silva pattern and stromal invasion level (i.e., cancers with Silva pattern C and deep stromal invasion).

CLDN18 has two isoforms, CLDN18 splice variant 1 (CLDN18.1) and CLDN18 splice variant 1 (CLDN18.2). CLDN18.1 is specific for epithelial cells in normal and cancerous lung tissues, and CLDN18.2 is expressed in normal gastric epithelial cells and in gastric, pancreatic, and esophageal cancer cells.^26^ The monoclonal antibody claudiximab (IMAB362, zolbetuximab) targets the membranous CLDN18.2 isoform^27^ and induces cancer cell apoptosis and immune effectors by activating antibody‐dependent cellular cytotoxicity (ADCC) and complement‐dependent cytotoxicity (CDC).^28^ Currently, several clinical trials have demonstrated improved efficacy among the subgroup of patients with high CLDN18 (M)‐expressing tumors.^27^, ^29^ CLDN18 is a promising therapeutic target in gastric and gastroesophageal adenocarcinoma due to its high expression on the surface of tumor cells. Our findings suggest that patients with CLDN18 (M)‐positive ECA may be candidates for CLDN18 (M)‐targeted therapy.

An interesting observation in this study was CLDN18 (N) immunoreactivity in ECAs. This detailed report provides the first description of a close association between CLDN18 (N) expression and ECA, independent of CLDN18 (M) expression in these tumors. In this study, CLDN18 (N) expression was found to be associated with Silva pattern A, which was associated with a good prognosis.

Studies have shown that claudins localize to the nucleus. When CLDNs are fused to a strong NLS sequence, the cytoplasmic fraction disappears, and the protein is completely localized in the nucleus.^30^ Despite the absence of the NLS sequence, claudins may utilize the PDZ domain or other mechanisms for nuclear transportation.^31^ Furthermore, claudin‐23 is highly expressed in the nucleus of pancreatic cancer cells as an intracellular signaling molecule.^32^ Recently, Yu Takahashi and colleagues demonstrated that nuclear expression of CLDN18 was independently associated with gastric cancers and could be a novel diagnostic marker for gastric cancer. CLDN18 (N) is a new immunohistochemical early diagnostic marker for well‐differentiated gastric adenocarcinoma. In Irene Coati's study, strong nuclear positivity was observed in 22.5% of primary esophagogastric adenocarcinomas.^19^ Similarly, in our study, among primary HPV‐associated ECA samples, 14 (28%) had nuclear positivity, and high nuclear CLDN18 expression was associated with the Silva pattern, with a higher incidence of positive expression in Silva pattern A cancers. These findings may indicate that CLDN18 translocates to the nucleus under certain cancerous conditions and might have a different function from CLDN18 (M).

In conclusion, we found CLDN18 (M) expression in ECAs, particularly in HPV‐independent ECAs, suggesting that CLDN18 may be a novel potential therapeutic target in ECA. Moreover, nuclear staining of CLDN18 is a new immunohistochemical marker for ECAs showing Silva pattern A, which have a good prognosis. Although shuttling of CLDN18 into the nucleus occurs and there is one putative NLS sequence in CLDN18,^13^ little is known about the mechanism that activates this event. Although the existing data support that membranous and nuclear CLDNs may serve not only as TJ transmembrane proteins but also as intracellular signaling molecules that interact with transcriptional regulators, further studies are needed to identify the pathophysiological function of intracellular translocation of CLDN18. In this study, we found not only membranous and nuclear localization of CLDN18 expression but also different subclonal patterns in ECA tissues. However, whether CLDN18 subclonal expression patterns are related to CLDN18 genetic alterations has not been explored. Furthermore, studies are needed to investigate the diagnostic, prognostic, or therapeutic usefulness of membranous and nuclear staining of CLDN18 and its subclonal patterns and to develop clinical tests for ECAs.

## AUTHOR CONTRIBUTIONS

Xiuzhen Du: Study design, data collection, data analysis, and manuscript writing. Yanjiao Hu: Pathological diagnosis and pathology review. Xiaoyu Ji: Data collection and results analysis. Qingmei Zheng: Research funding support and manuscript proofreading. Lei Sui: Pathological data collection. Kejuan Song: Study conception and clinical data collection. Teng Lv: Clinical data collection. Yulong Chen: Clinical data collection. Han Zhao: Immunohistochemical staining. Shuzhen Dai: Guidance for the study design. Peng Zhao: Pathological diagnosis and pathology review. Qin Yao: Study design and conception, manuscript editing and proofreading, and acquisition of research funding. All authors have read and approved the final manuscript.

## FUNDING INFORMATION

The study received financial support from Science and Technology Bureau of Qingdao West Coast New Area (grant number 2019‐55).

## CONFLICTS OF INTEREST

The authors declare no conflicts of interest.

## ETHICS STATEMENT

Our study was conducted in accordance with the principles of the Declaration of Helsinki and approved by the Institutional Review Committee of The Affiliated Hospital of Qingdao University. The clinicopathological information was collected from the patients' medical records and pathology reports. All data were anonymized prior to histology and immunohistochemistry.

## Data Availability

Information about the data provided in the article is available.
